# Incidence and risk factors for venous thromboembolism in the Cancer-VTE Registry pancreatic cancer subcohort

**DOI:** 10.1007/s00535-023-02033-3

**Published:** 2023-09-07

**Authors:** Takuji Okusaka, Akio Saiura, Kazuaki Shimada, Masafumi Ikeda, Tatsuya Ioka, Tetsuya Kimura, Jun Hosokawa, Atsushi Takita, Mari S. Oba

**Affiliations:** 1 Department of Hepatobiliary and Pancreatic Oncology, National Cancer Center Hospital, 5-1-1 Tsukiji, Chuo-ku, Tokyo 104-0045 Japan; 2 Department of Hepatobiliary-Pancreatic Surgery, Juntendo University Graduate School of Medicine, Bunkyo-ku, Tokyo Japan; 3 Department of Hepatobiliary and Pancreatic Oncology, National Cancer Center Hospital East, Kashiwa, Japan; 4 Oncology Center, Yamaguchi University Hospital, Ube, Japan; 5 Primary Medical Science Department, Daiichi Sankyo Co., Ltd., Chuo-ku, Tokyo Japan; 6 Data Intelligence Department, Daiichi Sankyo Co., Ltd., Shinagawa-ku, Tokyo Japan; 7 Department of Medical Statistics, Toho University, Ota-ku, Tokyo Japan; 8 Department of Clinical Data Science, Clinical Research & Education Promotion Division, National Center of Neurology and Psychiatry, Kodaira, Japan

**Keywords:** Incidence, Risk factors, Venous thromboembolism, Pancreatic cancer, Mortality

## Abstract

**Background:**

This substudy of the Cancer-VTE Registry estimated venous thromboembolism (VTE) incidence and risk factors in pancreatic cancer patients.

**Methods:**

The Cancer-VTE Registry was an observational study that collected VTE data from patients with solid tumors across Japan. We measured baseline VTE prevalence, and at 1-year follow-up, the cumulative incidence of symptomatic and composite VTE (symptomatic VTE and incidental VTE requiring treatment), bleeding, cerebral infarction/transient ischemic attack (TIA)/systemic embolic event (SEE), and all-cause death.

**Results:**

Of 1006 pancreatic cancer patients, 86 (8.5%) had VTE at baseline, and seven (0.7%) had symptomatic VTE. Significant risk factors of baseline VTE were Eastern Cooperative Oncology Group performance status (ECOG PS) of 1, body mass index (BMI) ≥ 25 kg/m^2^, history of VTE, D-dimer > 1.2 µg/mL, and hemoglobin < 10 g/dL. At 1-year follow-up, the cumulative incidence of events was higher for pancreatic cancer vs other cancers. Pancreatic cancer patients with VTE vs those without VTE had significantly higher incidences of bleeding, cerebral infarction/TIA/SEE, and all-cause death. No significant risk factors for composite VTE were identified.

**Conclusions:**

The cumulative incidence of composite VTE during cancer treatment was higher in pancreatic cancer than in other cancer types. Some risk factors for VTE prevalence at cancer diagnosis were identified. Although VTE prevalence at cancer diagnosis did not predict the subsequent 1-year incidence of composite VTE, it was a significant predictor of other events such as all-cause death in pancreatic cancer patients.

**Trial registration:**

UMIN Clinical Trials Registry; UMIN000024942

**Supplementary Information:**

The online version contains supplementary material available at 10.1007/s00535-023-02033-3.

## Introduction

Cancer-associated thrombosis, which includes both arterial and venous thromboembolism (VTE), is a significant complication. VTE is reported as the second most common cause of death in cancer patients [1]. The prognosis is worse for cancer patients with concomitant VTE compared with those without VTE.

Compared with other ethnic groups, Asian populations generally have a lower frequency of VTE, and VTE among Asian patients is more often asymptomatic than symptomatic [1, 2]. The Japanese Society of Cardiology first published guidelines for the diagnosis, treatment, and prevention of pulmonary embolism (PE) and deep vein thrombosis (DVT) in 2004 and the guidelines were subsequently revised in 2009 and 2017 [3]. Since then, VTE has been diagnosed more frequently in Japan, partly because of improvements in the recognition and diagnosis of the disease [4], resulting in increased interest in VTE in cancer patients.

Pancreatic cancer is the fourth leading cause of cancer-related death in Japan. The prognosis of pancreatic cancer is poor, with only 8.5% of patients remaining alive 5 years after diagnosis [5]. Various assessments, such as Khorana, Vienna CATS, PROTECHT, and CONKO VTE, have been developed to predict the risk of VTE, all of which indicate that pancreatic cancer is among the most prothrombotic malignancies [6, 7]. Reportedly, the incidence of VTE can be four times higher in pancreatic cancer than in other cancer types [8].

Currently, there is a lack of comprehensive and prospective research on VTE in Japanese patients with pancreatic cancer. The incidence and impact of VTE complications, as well as the underlying risk factors, have yet to be thoroughly studied in this population.

The Cancer-VTE Registry was a Japanese, multicenter, prospective registry study that aimed to collect data on VTE in patients with colorectal, lung, stomach, breast, gynecological, or pancreatic cancer [9–11]. The present substudy of the Cancer-VTE Registry aimed to estimate the incidence and risk factors of VTE in patients with pancreatic cancer in the Cancer-VTE Registry. Additionally, we investigated how concurrent VTE affects mortality in patients with pancreatic cancer.

## Methods

### Study design

The rationale and design of the Cancer-VTE Registry (UMIN000024942) have been previously reported [9, 10]. Briefly, the Cancer-VTE Registry was a nationwide, large-scale multicenter observational study undertaken in Japan between March 2017 and September 2020, with a 1-year follow-up.

All patients had undergone VTE screening via lower extremity venous ultrasonography or computed tomography (CT) angiography 2 months before enrollment. The Japan Society of Ultrasonics in Medicine guidelines were used as the standard for venous ultrasonography of the lower extremity [12]; however, this could be substituted by CT angiography of the lower extremity. At the physician’s discretion, PE was confirmed via contrast CT or other diagnostic imaging tests. Additionally, if the D-dimer value measured after the cancer diagnosis was ≤ 1.2 µg/mL [13], VTE screening was not necessarily required, and the patient was considered to have no VTE.

The cutoffs for platelet count, hemoglobin, and white blood cell count used in this study were based on the Khorana Risk Score for Venous Thromboembolism in Cancer Patients [14].

### Patients

Enrolled patients included hospitalized patients or outpatients aged ≥ 20 years with a diagnosis of pancreatic cancer, confirmed stage II–IV, with planned initiation of cancer therapy (patients were enrolled regardless of whether they had primary or recurrent cancer), Eastern Cooperative Oncology Group performance status (ECOG PS) of 0–1, and a life expectancy of ≥ 3 months.

The main exclusion criteria were the presence of active double cancer, patients who were difficult to follow-up, or patients the investigator deemed inappropriate to participate in the study.

### Outcomes

The outcome was the prevalence of VTE at baseline, including symptomatic/incidental PE and symptomatic/asymptomatic DVT (both proximal and distal), among patients with pancreatic cancer.

At 1-year follow-up, the incidence and cumulative incidence of symptomatic VTE, composite VTE (symptomatic VTE events and incidental [asymptomatic] VTE events requiring treatment), bleeding (major or clinically relevant non-major bleeding), cerebral infarction/transient ischemic attack (TIA)/systemic embolic event (SEE), and all-cause death were calculated. The following events indicated incidental VTE requiring treatment: (1) incidentally discovered asymptomatic VTE for which patients started treatment during the follow-up period; or (2) asymptomatic VTE that was detected at baseline screening, had remained untreated, and for which patients initiated therapy during the follow-up period based on clinical indication, such as the onset of an exacerbation. The incidence of each event was compared between patients with and those without VTE at baseline and patients with other cancer types (i.e., colorectal, lung, stomach, breast, and gynecologic cancers, including endometrial, cervical, ovarian, fallopian tube, and peritoneal cancers).

### Statistical analysis

Details of the statistical analysis, including sample size calculations, have been reported previously [9, 10]. For this substudy, the incidence of VTE at baseline was calculated as a proportion. Univariable and multivariable logistic regression analyses were conducted to detect the risk factors for VTE at baseline.

The cumulative incidence rate of each event during the follow-up period among patients with pancreatic cancer and the other five cancer types enrolled in the Cancer-VTE Registry were estimated, and hazard ratios (HRs) and 95% confidence intervals (CIs) were calculated using Fine and Gray’s model (for VTE, bleeding, and cerebral infarction/TIA/SEE) or the Cox model (for all-cause death). *P* values were calculated using Gray’s test (for VTE, bleeding, and cerebral infarction/TIA/SEE) or the log-rank test (for all-cause death). The same methodology was used to determine the between-group differences of these events during follow-up by VTE status using all-cause death as a competing event. Risk factors for composite VTE during the follow-up period were explored using the Fine and Gray models, with all-cause death as a competing event. The data analysis was conducted using SAS software version 9.4 (SAS Institute Inc., Cary, NC, USA).

### Ethical approval

The ethics committee at each participating institution approved the protocol. The study adhered to the Declaration of Helsinki and the Ethical Guidelines for Medical Science Studies on Human Subjects by the Japanese Ministry of Education, Culture, Sports, Science and Technology and the Ministry of Health, Labour and Welfare. All patients provided written informed consent.

## Results

The prevalence of VTE at baseline among patients with pancreatic cancer from the Cancer-VTE Registry is summarized in Table 1. Of the 1006 patients with pancreatic cancer in the Cancer-VTE Registry (*N* = 9630 in total), 86 (8.5%) patients had VTE at baseline. Most patients with VTE at baseline had asymptomatic VTE (*n* = 79; 7.9%), and the remaining patients had symptomatic VTE (*n* = 7; 0.7%). Ten patients (1.0%) had PE (with or without DVT), and all PE cases were asymptomatic. Most patients (*n* = 84; 8.3%) had DVT.

**Table 1:** Summary of VTE prevalence at baseline in patients with pancreatic cancer

*n* (%)	All	Symptomatic	Asymptomatic
All VTE	86 (8.5)	7 (0.7)	79 (7.9)
PE (with/without DVT)	10 (1.0)	0 (0.0)	10 (1.0)
DVT (with/without PE)	84 (8.3)	7 (0.7)	77 (7.7)
Proximal DVT	17 (1.7)	4 (0.4)	13 (1.3)
Distal DVT	67 (6.7)	3 (0.3)	64 (6.4)

Data are calculated based on *N* = 1006. *DVT* deep vein thrombosis, *PE* pulmonary embolism, *VTE* venous thromboembolism

Table 2 shows the characteristics of pancreatic cancer patients with and without VTE at baseline. Overall, 56.2% of patients were male, and the mean age was 67.6 years. Most patients had stage IV pancreatic cancer, which was present in 41.5% of patients. The most common cancer subtype was exocrine neoplasms (91.9%), with most cases being invasive ductal carcinoma (86.4%). When comparing the backgrounds of patients with and without VTE at baseline, more patients with VTE at baseline had distant metastasis, stage IV, and ECOG PS 1 cancer than those without VTE at baseline. There were no differences in cancer subtypes between patients with and without VTE at baseline, although the number of patients with a cancer subtype other than intraductal papillary neoplasm or invasive ductal carcinoma was small. Additionally, patients with VTE had higher oral anticoagulant use and D-dimer level than those without VTE (Table 2).

**Table 2:** Patient characteristics

	Pancreatic cancer patients (*n* = 1006)	With VTE at baseline (*n* = 86)	Without VTE at baseline (*n* = 920)
Male sex, *n* (%)	565 (56.2)	36 (41.9)	529 (57.5)
Age, years			
Mean (median) ± SD	67.6 (69.0) ± 9.8	70.6 (72.0) ± 9.6	67.3 (69.0) ± 9.7
≥ 65, *n* (%)	680 (67.6)	69 (80.2)	611 (66.4)
BMI, kg/m^2^			
Mean (median) ± SD	21.8 (21.6) ± 3.3	22.0 (21.9) ± 3.3	21.8 (21.5) ± 3.4
≥ 25, *n* (%)	147 (14.6)	17 (19.8)	130 (14.1)
Primary cancer, *n* (%)	982 (97.6)	83 (96.5)	899 (97.7)
With lymph-node metastasis, *n* (%)	365 (36.3)	43 (50.0)	322 (35.0)
With distant metastasis, *n* (%)	401 (39.9)	61 (70.9)	340 (37.0)
Cancer stage, *n* (%)			
II	384 (38.2)	17 (19.8)	367 (39.9)
III	205 (20.4)	9 (10.5)	196 (21.3)
IV	417 (41.5)	60 (69.8)	357 (38.8)
ECOG PS			
0	738 (73.4)	41 (47.7)	697 (75.8)
1	268 (26.6)	45 (52.3)	223 (24.2)
Cancer subtype, *n* (%)			
Exocrine neoplasms	925 (91.9)	80 (93.0)	845 (91.8)
Serous cystadenocarcinoma	1 (0.1)	0 (0.0)	1 (0.1)
Mucinous cystic neoplasms	3 (0.3)	0 (0.0)	3 (0.3)
Intraductal papillary neoplasms	46 (4.6)	2 (2.3)	44 (4.8)
Invasive ductal carcinomas	869 (86.4)	78 (90.7)	791 (86.0)
Acinar cell adenocarcinoma	6 (0.6)	0 (0.0)	6 (0.7)
Neuroendocrine tumors	7 (0.7)	0 (0.0)	7 (0.8)
NET G1/G2	6 (0.6)	0 (0.0)	6 (0.7)
NEC	1 (0.1)	0 (0.0)	1 (0.1)
Other	73 (7.3)	6 (7.0)	67 (7.3)
History of VTE	12 (1.2)	8 (9.3)	4 (0.4)
Bed rest for 4 days or more	6 (0.6)	0 (0.0)	6 (0.7)
DOAC or warfarin use^a^, *n* (%)	82 (8.2)	44 (51.2)	38 (4.1)
D-dimer, µg/mL			
Mean (median) ± SD	2.0 (0.8) ± 4.1	8.7 (4.9) ± 10.6	1.3 (0.8) ± 1.9
> 1.2, *n* (%)	311 (30.9)	73 (84.9)	238 (25.9)
Platelet count, × 10^9^/L			
Mean (median) ± SD	236 (224) ± 83	243 (220) ± 86	236 (224) ± 83
≥ 350, *n* (%)	75 (7.5)	11 (12.8)	64 (7.0)
Hb, g/dL			
Mean (median) ± SD	12.8 (12.8) ± 1.6	12.0 (12.3) ± 1.9	12.9 (12.9) ± 1.5
< 10, *n* (%)	35 (3.5)	14 (16.3)	21 (2.3)
WBC count, × 10^9^/L			
Mean (median) ± SD	6.4 (5.8) ± 4.4	8.7 (6.4) ± 12.7	6.1 (5.8) ± 2.2
> 11, *n* (%)	28 (2.8)	8 (9.3)	20 (2.2)
CrCL, mL/min			
Mean (median) ± SD	78 (74) ± 26	76 (73) ± 27	79 (75) ± 26
≤ 50, *n* (%)	83 (8.3)	8 (9.3)	75 (8.2)

Percentages shown in the table calculated based on the total in each column unless otherwise specified

^a^Oral anticoagulant treatment that started before enrollment

*BMI* body mass index, *CrCL* creatinine clearance, *DOAC* direct oral anticoagulant, *ECOG PS* Eastern Cooperative Oncology Group performance status, *G* grade, *Hb* hemoglobin, *NEC* neuroendocrine carcinoma, *NET* neuroendocrine tumor, *SD* standard deviation, *VTE* venous thromboembolism, *WBC* white blood cell

We analyzed the outcomes during the follow-up period for patients with (*n* = 86) and without (*n* = 920) VTE at baseline, stratified by direct oral anticoagulant (DOAC) or warfarin use (Supplementary Table 1). In the 44 patients with VTE who received DOACs or warfarin and 42 patients with VTE who did not, the incidence of VTE during the follow-up period was low (0–2) in both groups, with no apparent difference between groups. The population that received DOAC or warfarin showed a suggested trend toward a higher incidence of bleeding*,* irrespective of with VTE at baseline or without.

In total, the proportions of bleeding, major bleeding, and clinically relevant non-major bleeding events were 3.8, 1.5, and 2.4%, respectively. Bleeding events were predominantly gastrointestinal in nature (e.g., gastrointestinal bleeding, melena, small intestinal hemorrhage, intra-abdominal hemorrhage, hematemesis, and gastric hemorrhage; Supplementary Table 2).

The multivariable analysis revealed that significant factors that correlated with VTE prevalence at baseline were ECOG PS 1 vs PS 0 (odds ratio [OR]: 1.85, 95% CI: 1.02–3.38; *p* = 0.044), body mass index (BMI) of ≥ 25 kg/m^2^ (OR: 2.20, 95% CI: 1.03–4.71; *p* = 0.042), history of VTE (OR: 135.40, 95% CI: 24.09–761.13; *p* < 0.001), D-dimer of > 1.2 μg/mL (OR: 24.80, 95% CI: 9.57–64.30;* p* < 0.001), and Hb of < 10 g/dL (OR: 3.64, 95% CI: 1.42–9.35;* p* = 0.007) (Table 3).

**Table 3:** Univariable and multivariable analysis of factors correlated with VTE prevalence at baseline

Items		*N*	Events, *n* (%)	Univariable			Multivariable		
				OR	95% CI	*p* value	OR	95% CI	*p* value
Sex	Male	565	36 (6.4)	Reference	–	–	Reference	–	–
	Female	441	50 (11.3)	1.88	1.20–2.94	0.006	1.52	0.85–2.72	0.157
Age, years	< 65	326	17 (5.2)	Reference	–	–	Reference	–	–
	≥ 65	680	69 (10.1)	2.05	1.19–3.55	0.010	1.48	0.73–2.99	0.279
Cancer stage	II	384	17 (4.4)	Reference	–	–	Reference	–	–
	III	205	9 (4.4)	0.99	0.43–2.27	0.984	1.22	0.45–3.28	0.694
	IV	417	60 (14.4)	3.63	2.08–6.34	< 0.001	1.73	0.83–3.58	0.142
ECOG PS	0	738	41 (5.6)	Reference	–	–	Reference	–	–
	1	268	45 (16.8)	3.43	2.19–5.38	< 0.001	1.85	1.02–3.38	0.044
BMI, kg/m^2^	< 18.5	144	14 (9.7)	1.28	0.69–2.38	0.429	1.61	0.71–3.64	0.256
	18.5 to < 25	710	55 (7.7)	Reference	–	–	Reference	–	–
	≥ 25	147	17 (11.6)	1.56	0.88–2.77	0.131	2.20	1.03–4.71	0.042
History of VTE	No	994	78 (7.8)	Reference	–	–	Reference	–	–
	Yes	12	8 (66.7)	23.49	6.92–79.73	< 0.001	135.40	24.09–761.13	< 0.001
D-dimer, μg/mL	≤ 1.2	675	9 (1.3)	Reference	–	–	Reference	–	–
	> 1.2	311	73 (23.5)	22.70	11.18–46.08	< 0.001	24.80	9.57–64.30	< 0.001
Platelet count, × 10^9^/L	< 350	813	66 (8.1)	Reference	–	–	Reference	–	–
	≥ 350	75	11 (14.7)	1.95	0.98–3.87	0.058	0.91	0.35–2.37	0.844
Hb, g/dL	≥ 10	853	63 (7.4)	Reference	–	–	Reference	–	–
	< 10	35	14 (40.0)	8.36	4.06–17.23	< 0.001	3.64	1.42–9.35	0.007
WBC count, × 10^9^/L	≤ 11	860	69 (8.0)	Reference	–	–	Reference	–	–
	> 11	28	8 (28.6)	4.59	1.95–10.80	0.001	3.06	0.89–10.51	0.075

The multivariable analysis used variables listed in this table as explanatory variables

*BMI* body mass index, *CI* confidence interval, *ECOG PS* Eastern Cooperative Oncology Group performance status, *Hb* hemoglobin, *OR* odds ratio, *VTE* venous thromboembolism, *WBC* white blood cell

To examine the characteristics of the event incidence in patients with pancreatic cancer, we compared it with the cumulative incidence of VTE in five other cancer types among patients enrolled in the Cancer-VTE Registry. The mean follow-up period was 315.7 days in patients with pancreatic cancer and 358.0 days in the five other cancer types.

**Fig. 1:**
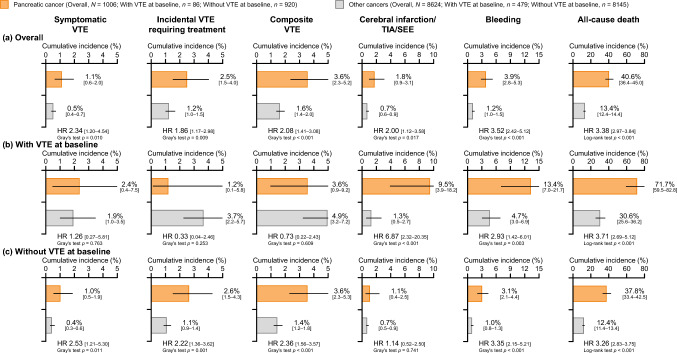
Cumulative incidence of events during the follow-up period (**a**) overall; (**b**) in patients with VTE at baseline screening; and (**c**) patients without VTE at baseline screening. The reference is the other five cancer types. “Other cancers” was a composite of cancers other than pancreatic cancer in the Cancer-VTE Registry (colorectal, lung, stomach, breast, and gynecologic cancer). *p* values in pancreatic cancer patients vs patients with the five other cancer types were calculated using Gray’s test for events other than all-cause death and the log-rank test for all-cause death. Error bars denote 95% CIs. *CI* confidence interval, *HR* hazard ratio, *SEE* systemic embolic event, *TIA* transient ischemic attack, *VTE* venous thromboembolism

Figure 1 shows the cumulative incidence of symptomatic VTE, incidental VTE requiring treatment, composite VTE, cerebral infarction/TIA/SEE, bleeding, and all-cause death in patients with pancreatic cancer vs patients with the other five types of cancer. During the 1-year follow-up period, the overall cumulative incidence of each event was higher in the pancreatic cancer subgroup than the five other cancers: symptomatic VTE (1.1% vs 0.5%; HR [95% CI], 2.34 [1.20–4.54], Gray’s test *p* = 0.010), incidental VTE requiring treatment (2.5% vs 1.2%; HR [95% CI] 1.86 [1.17–2.98]; Gray’s test *p* = 0.009), composite VTE (3.6% vs 1.6%; HR [95% CI], 2.08 [1.41–3.08], Gray’s test *p* < 0.001), cerebral infarction/TIA/SEE (1.8% vs 0.7%; HR [95% CI] 2.00 [1.12–3.58]; Gray’s test *p* = 0.017), bleeding (3.9% vs 1.2%; HR [95% CI] 3.52 [2.42–5.12]; Gray’s test *p* < 0.001), and all-cause death (40.6% vs 13.4%; HR [95% CI], 3.38 [2.97–3.84], log-rank test *p* < 0.001) (Fig. 1a). Among patients with VTE at baseline, there was no significant difference between pancreatic and other cancers in the incidence of symptomatic VTE, incidental VTE requiring treatment, and composite VTE. However, the incidence of cerebral infarction/TIA/SEE (9.5% vs 1.3%; HR [95% CI] 6.87 [2.32–20.35]; Gray’s test *p* < 0.001), bleeding (13.4% vs 4.7%; HR [95% CI] 2.93 [1.42–6.01]; Gray’s test *p* = 0.003), and all-cause death (71.7% vs 30.6%; HR [95% CI] 3.71 [2.69–5.12]; log-rank test *p* < 0.001) was significantly higher among patients with pancreatic vs other types of cancer and VTE at baseline (Fig. 1b). For patients without VTE at baseline, the incidence of symptomatic VTE, incidental VTE requiring treatment, composite VTE, bleeding, and all-cause death was significantly higher among patients with pancreatic cancer than those with other cancers, except for cerebral infarction/TIA/SEE (Fig. 1c).

**Fig. 2:**
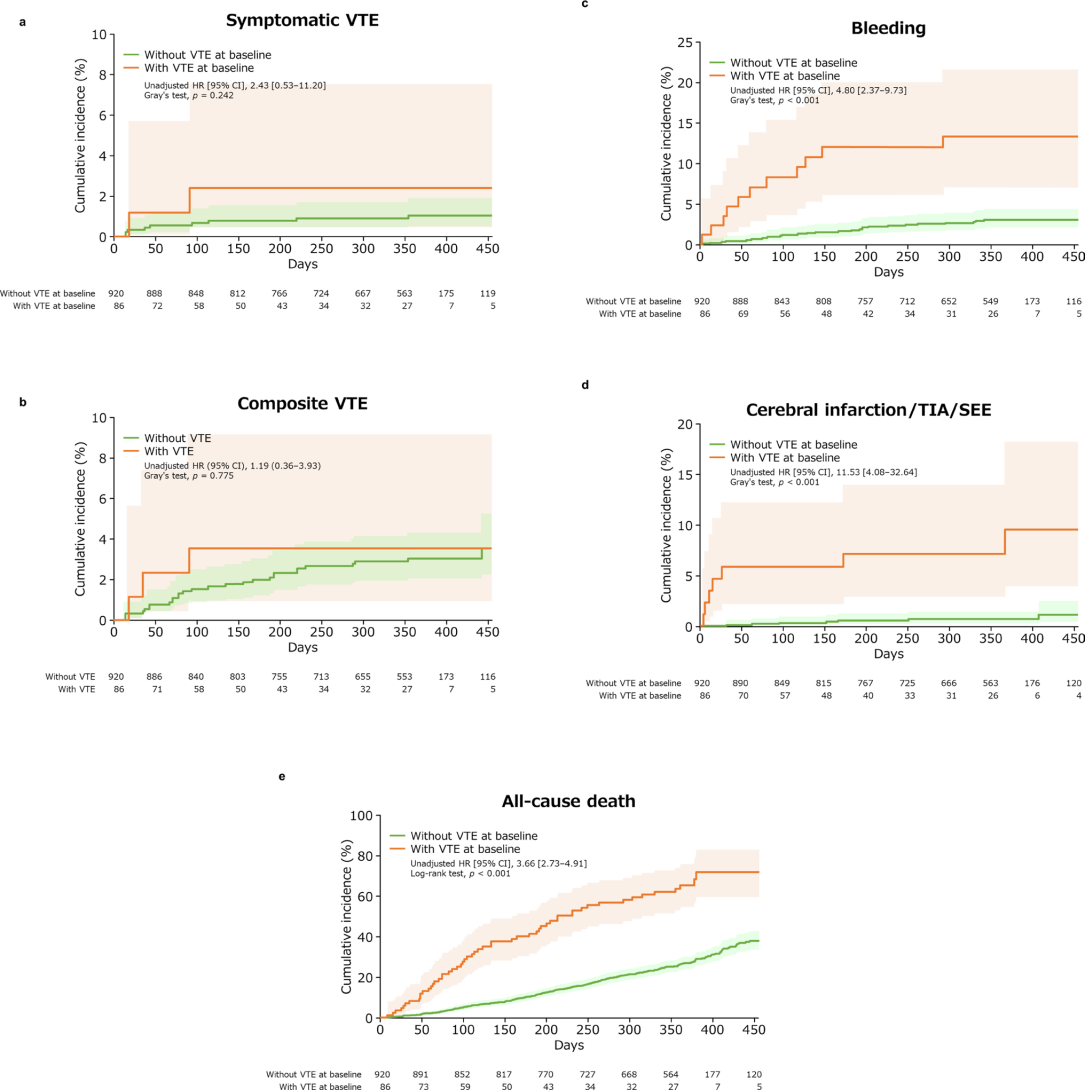
Cumulative incidence of (**a**) symptomatic VTE; (**b**) composite VTE; (**c**) bleeding events; (**d**) cerebral infarction/TIA/SEE; (**e**) all-cause death events (time-to-event analysis) in patients with and without VTE at baseline. *p* values were calculated using Gray’s test (**a**–**d**) or log-rank test (**e**). Lightly shaded areas represent 95% CIs. *CI* confidence interval, *HR* hazard ratio, *SEE* systemic embolic event, *TIA* transient ischemic attack, *VTE* venous thromboembolism

Figure 2 shows the cumulative incidence of symptomatic VTE, composite VTE, bleeding, cerebral infarction/TIA/SEE, and all-cause death in patients with and without VTE at baseline. The cumulative incidence of symptomatic VTE (unadjusted HR [95% CI], 2.43 [0.53–11.20], Gray’s test *p* = 0.242; Fig. 2a) and that of composite VTE (1.19 [0.36–3.93], Gray’s test *p* = 0.775; Fig. 2b) were not significantly higher among pancreatic cancer patients with VTE vs those without VTE. However, patients with VTE vs those without VTE had significantly higher incidences of bleeding (4.80 [2.37–9.73], Gray’s test *p* < 0.001; Fig. 2c), cerebral infarction/TIA/SEE (11.53 [4.08–32.64], Gray’s test *p* < 0.001; Fig. 2d) and all-cause death (3.66 [2.73–4.91], log-rank test *p* < 0.001; Fig. 2e).

Table 4 shows univariable and multivariable analyses of risk factors for composite VTE during the follow-up period. Multivariable analysis showed that the HRs of female sex, age ≥ 65 years, cancer stage III or IV, and D-dimer of > 1.2 µg/mL were greater than 1, but none of the differences reached statistical significance.

**Table 4:** Univariable and multivariable analysis of risk factors for composite VTE during the follow-up period

Items		*N*	Events, *n* (%)	Univariable			Multivariable		
				HR	95% CI	*p* value	HR	95% CI	*p* value
Sex	Male	565	14 (2.5)	Reference	–	–	Reference		
	Female	441	17 (3.9)	1.55	0.76–3.14	0.225	1.45	0.69–3.05	0.324
Age, years	< 65	326	5 (1.5)	Reference	–	–	Reference		
	≥ 65	680	26 (3.8)	2.54	0.98–6.60	0.055	2.49	0.93–6.67	0.070
Cancer stage	II	384	8 (2.1)	Reference	–	–	Reference		
	III	205	8 (3.9)	1.92	0.72–5.14	0.196	2.00	0.75–5.40	0.169
	IV	417	15 (3.6)	1.75	0.74–4.13	0.201	1.66	0.67–4.11	0.276
VTE prevalence at baseline	No	920	28 (3.0)	Reference	–	–	Reference		
	Yes	86	3 (3.5)	1.19	0.36–3.93	0.779	0.66	0.18–2.38	0.521
D-dimer, μg/mL	≤ 1.2	675	16 (2.4)	Reference	–	–	Reference		
	> 1.2	311	15 (4.8)	2.10	1.04–4.25	0.039	2.03	0.96–4.31	0.064

The multivariable analysis used variables listed in this table as explanatory variables

*CI* confidence interval, *HR* hazard ratio, *VTE* venous thromboembolism

## Discussion

Prior to our study, there was a lack of extensive research on VTE in Japanese pancreatic cancer patients. The occurrence of VTE complications, its correlation with mortality, and the risk factors for VTE had not been adequately studied. Our novel study aimed to address these gaps by comprehensively investigating Japanese pancreatic cancer patients, and assessing the impact of the presence or absence of concomitant VTE on events during cancer treatment.

Among Japanese patients with pancreatic cancer enrolled in the Cancer-VTE Registry, the VTE prevalence at the baseline screening was 8.5%, of which the prevalence of symptomatic VTE was low at 0.7%. Risk factors of VTE at baseline among pancreatic cancer patients were ECOG PS, BMI, D-dimer, Hb, and history of VTE. The prevalence of VTE in this cohort of patients with pancreatic cancer (8.5%) was higher than that overall (5.9%), and pancreatic cancer was the cancer type with the highest VTE frequency in the Cancer-VTE Registry compared with stomach (6.9%), colorectal (6.4%), gynecologic (5.5%), lung (5.1%), and breast (2.0%) cancer [10].

During cancer therapy (1-year follow-up period), the cumulative incidence was 1.1% for symptomatic VTE, 2.5% for incidental VTE requiring treatment, 3.6% for composite VTE, 1.8% for cerebral infarction/TIA/SEE, and 40.6% for all-cause death for patients with pancreatic cancer, all of which were also higher than those of patients with other cancer types from the Cancer-VTE Registry. These results were similar to those reported in non-Japanese studies [15, 16].

VTE is generally understood to be less common in Asian people than in Caucasians [2, 17]. Indeed, the present findings are consistent with previous reports, as the incidence of VTE in our study (3.6%) was lower than that reported at 12 months in pancreatic cancer patients in observational studies in France (19.2%) and the United States (10.7%) [18, 19].

Comparing the findings with other Japanese studies, the prevalence and incidence of VTE in the present study (8.5 and 3.6%, respectively) were generally lower than those reported in other Japanese studies [20–22]. However, it must be noted that these studies had important differences compared with the current study, including the study design and diagnostic criteria for VTE.

VTE prevalence at baseline was not determined as a statistically significant risk factor for composite VTE during the follow-up period, which is different from the results of the Cancer-VTE Registry subanalysis in colorectal and lung cancers [23, 24]. This difference may be attributable to the poor prognosis of pancreatic cancer patients with VTE at baseline. In fact, the incidences of all-cause death in pancreatic cancer patients with VTE at baseline was very high (71.7%), which leads us to speculate that death may have occurred before recurrent VTE and recurrent VTE could not have been detected during the 1-year follow-up. Thus, our results indicate that pancreatic cancer patients with VTE at the time of cancer diagnosis are not at low risk of VTE recurrence, but rather represent a population with a poor prognosis.

Pancreatic cancer patients with VTE at baseline had a higher risk of death (unadjusted HR [95% CI], 3.66 [2.73–4.91]), but the severity of VTE at baseline in most cases was mild, including asymptomatic (*n* = 79/86) and distal DVT (*n* = 67/84). Relationships between VTE and death may be attributable to similarities in the mechanisms of coagulation and cancer metastasis. For example, tissue factor (TF) is the initiator of the extrinsic coagulation cascade, but it is also present in cancer cells [25]. Hematogenous metastasis involves the release of microvesicles containing TF [26]. TF is highly expressed in pancreatic cancer [27], and this is thought to contribute to the higher expression of VTE in pancreatic cancer. In a previous study of resectable pancreatic cancer, death occurred 2.9–3.7 times more frequently in patients with VTE [28], which was consistent with the present study findings. Thus, the presence of VTE may not only induce thrombosis and directly increase mortality but may also be reflective of cancer progression and worsening.

The study revealed that the risk factors for VTE at the time of cancer diagnosis were poor ECOG PS, BMI ≥ 25 kg/m^2^, previous history of VTE, D-dimer > 1.2 µg/mL, and Hb < 10 g/dL, and this was generally consistent with previous studies. A Taiwanese study of patients with lung, gastric, and pancreatic cancer and patients with lymphoma reported that either very low or very high BMI (male BMI ≤ 23.940 and > 31.259 kg/m^2^, female BMI ≤ 19.424 and > 31.538 kg/m^2^) and anemia (male Hb ≥ 8.785 and < 13.435 g/dL, female Hb ≥ 6.963 and < 11.737 g/dL), which differed by sex, were significant risk factors [29]. A Japanese study of VTE among patients with pancreatic cancer reported that significantly more patients with VTE had a BMI > 25 kg/m^2^, and a significantly higher proportion of patients had undergone chemotherapy, particularly with gemcitabine or paclitaxel [22]. Another recent study of Japanese patients with pretreated advanced pancreatic cancer reported that high levels of D-dimer, prothrombin fragment 1 + 2, fibrin degradation product, and thrombin/antithrombin III complex were associated with VTE occurrence [20]. Among patients surgically treated for pancreatic cancer, a high BMI (≥ 30 kg/m^2^), previous anticoagulation treatment, and disease recurrence were risk factors for VTE [28].

The frequency of VTE at the time of cancer diagnosis was higher in patients with a history of VTE in the present study, which was consistent with previous studies [30, 31]. This suggests that collecting information on episodes related to VTE is important during the interview at the time of cancer diagnosis. In contrast, no significant factors were identified as risk factors for composite VTE during the follow-up period, but factors with HR ≥ 2 were age ≥ 65 years, cancer stage III, and D-dimer > 1.2 µg/mL at baseline. The incidence of composite VTE during the follow-up period was low (31 events), which may have been insufficient to perform multivariate analysis.

This study showed that patients with pancreatic cancer had a higher incidence of bleeding events than patients with the other five cancer types (HR [95% CI], 3.52 [2.42–5.12]). Bleeding events in this study were collected according to the definition of bleeding severity only [9–11], without considering the cause (e.g., drug administration or surgery). Although a detailed analysis of bleeding could not be performed, it was suggested that pancreatic cancer patients might be more prone to bleeding than other cancers. Additionally, pancreatic cancer is a cancer type associated with a high incidence of VTE; therefore, it is important to carefully consider the risk–benefit balance when anticoagulation therapy is initiated to treat VTE.

In this study, patients with VTE at cancer diagnosis, even those with asymptomatic VTE, had a higher incidence of cerebral infarction/TIA/SEE during the follow-up period (unadjusted HR [95% CI], 11.53 [4.08–32.64]). Trousseau syndrome is a known condition in which cancer patients exhibit cerebral infarction, and one of the mechanisms is thought to involve increased TF expression by tumor cells [32]. VTE and arterial thromboembolism in cancer patients are sometimes collectively referred to as cancer-associated thrombosis. Plasma TF levels have been reported to be an independent predictor of cancer-associated thrombosis in patients with pancreatic cancer [33]. However, the direct relationship between VTE and cerebral infarction/TIA/SEE in patients with pancreatic cancer remains unclear and is a subject for future studies.

In Japan, pancreatic cancer patients have a higher incidence of VTE than other cancers. It was also shown that concomitant VTE has a poor prognosis. Because asymptomatic VTE is the main component, it suggests the importance of proactively evaluating VTE risk factors at the time of cancer diagnosis and performing VTE screening if necessary. In the Japanese Clinical Practice Guidelines for Pancreatic Cancer [34], VTE was mentioned for the first time in the 2022 edition. We hope that the results of this study will be useful for future guideline updates.

This study had some limitations. First, patients with more advanced cancer (life expectancy ≤ 3 months and ECOG PS ≥ 2) were not included in this study; thus, the findings cannot be generalized to these patients. Moreover, the population included patients who had undergone curative resection, and the frequency of events may differ between such patients and those with active cancer. Second, VTE treatment at the time of enrollment was at the attending physician’s discretion. Anticoagulants (warfarin or direct oral anticoagulants) may have been used for unrelated comorbidities, such as atrial fibrillation, but may also have been used for treating asymptomatic VTE, which might have led to fewer VTE events in the follow-up period. Third, we did not collect data on the cause of bleeding, tumor markers such as CA19-9, or D-dimer during the follow-up period or at the time of event occurrence. Any correlation between D-dimer values and the occurrence of events during the follow-up period unclear. Fourth, assessment of the impact of anticoagulants on events in the follow-up period was done only stratified by the status of DOAC or warfarin use at baseline. The purpose and the actual dosage of anticoagulants administered during the follow-up period were not considered. It should be noted that this is a crude analysis and confounding may occur. Finally, underestimation of the prevalence of VTE at baseline cannot be ruled out.

In conclusion, in this substudy of the Cancer-VTE Registry, 8.5% of patients with pancreatic cancer had VTE at the time of cancer diagnosis, and poor ECOG PS, high BMI, history of VTE, high D-dimer, and low hemoglobin were identified as a risk factor. Additionally, the cumulative incidence of composite VTE during cancer treatment was higher in patients with pancreatic cancer than in those with other cancer types. Although the presence of VTE at the time of cancer diagnosis was not a significant factor to indicate the subsequent 1-year incidence of VTE, it was a significant prognostic factor for other events, such as all-cause death, in patients with pancreatic cancer.

### Supplementary Information

Below is the link to the electronic supplementary material.

Supplementary file1 (DOCX 26 KB)

## Data Availability

The anonymized data underlying the results presented in this manuscript may be made available to researchers upon submission of a reasonable request to the corresponding author. The decision to disclose the data will be made by the corresponding author and the funder, Daiichi Sankyo Co., Ltd. The data disclosure can be requested for 36 months from the article publication.

## Abbreviations

BMI: Body mass index
CI: Confidence interval
CrCL: Creatinine clearance
CT: Computed tomography
DOAC: Direct oral anticoagulant
DVT: Deep vein thrombosis
ECOG PS: Eastern Cooperative Oncology Group performance status
Hb: Hemoglobin
HR: Hazard ratio
OR: Odds ratio
PE: Pulmonary embolism
SEE: Systemic embolic event
TF: Tissue factor
TIA: Transient ischemic attack
VTE: Venous thromboembolism
