# Supplementary material for: Incidence and risk factors for venous thromboembolism in the Cancer-VTE Registry pancreatic cancer subcohort
Source: J Gastroenterol. 2023 Sep 7;58(12):1261–71. doi: 10.1007/s00535-023-02033-3 (PMC10657787; doi:10.1007/s00535-023-02033-3)
Supplement: Supplementary file 1 — Supplementary file1 (DOCX 26 KB) [file 535_2023_2033_MOESM1_ESM.docx]

**Supplemental Table 1.** Outcomes by presence or absence of VTE prevalence at baseline and by use of oral anticoagulant at baseline (pancreatic cancer patients, n = 1006)

|  | DOAC or warfarin use^a^ | *N* | Symptomatic  VTE | | Incidental VTE  requiring treatment | | Composite  VTE | | Cerebral infarction/ TIA/SEE | | Bleeding | | All-cause death | |
| --- | --- | --- | --- | --- | --- | --- | --- | --- | --- | --- | --- | --- | --- | --- |
|  |  |  | Event  *n* | Incidence  % (95% CI) | Event  *n* | Incidence  % (95% CI) | Event  *n* | Incidence  % (95% CI) | Event  *n* | Incidence  % (95% CI) | Event  *n* | Incidence  % (95% CI) | Event  *n* | Incidence  % (95% CI) |
| With VTE at baseline  (*n* = 86) | Yes | 44 | 1 | 2.3  (0.1–12.0) | 0 | 0.0  (0.0–8.0) | 1 | 2.3  (0.1–12.0) | 6 | 13.6  (5.2–27.4) | 7 | 15.9  (6.6–30.1) | 30 | 68.2  (52.4–81.4) |
|  | No | 42 | 1 | 2.4  (0.1–12.6) | 1 | 2.4  (0.1–12.6) | 2 | 4.8  (0.6–16.2) | 1 | 2.4  (0.1–12.6) | 4 | 9.5  (2.7–22.6) | 24 | 57.1  (41.0–72.3) |
| Without VTE at baseline  (*n* = 920) | Yes | 38 | 1 | 2.6  (0.1–13.8) | 1 | 2.6  (0.1–13.8) | 2 | 5.3  (0.6–17.7) | 0 | 0.0  (0.0–9.3) | 3 | 7.9  (1.7–21.4) | 10 | 26.3  (13.4–43.1) |
|  | No | 882 | 8 | 0.9  (0.4–1.8) | 19 | 2.2  (1.3–3.3) | 26 | 2.9  (1.9–4.3) | 7 | 0.8  (0.3–1.6) | 24 | 2.7  (1.8–4.0) | 249 | 28.2  (25.3–31.3) |

^a^Oral anticoagulant treatment that started before enrollment.

*CI* confidence interval, *DOAC* direct oral anticoagulant, *SEE* systemic embolic event, *TIA* transient ischemic attack, *VTE* venous thromboembolism

**Supplementary Table 2**. Details of bleeding events (*n* = 1006)

|  | **Bleeding**  ***n* (%)** | **Major bleeding**  ***n* (%)** | **Clinically relevant non-major bleeding**  ***n* (%)** |
| --- | --- | --- | --- |
| **Total** | **38 (3.8)** | **15 (1.5)** | **24 (2.4)** |
| **Neoplasms benign, malignant and unspecified (incl cysts and polyps)** | **1 (<0.1)** | **1 (<0.1)** | **0** |
| Tumor hemorrhage | 1 (<0.1) | 1 (<0.1) | 0 |
| **Blood and lymphatic system disorders** | **1 (<0.1)** | **0** | **1 (<0.1)** |
| Disseminated intravascular coagulation | 1 (<0.1) | 0 | 1 (<0.1) |
| **Eye disorders** | **1 (<0.1)** | **0** | **1 (<0.1)** |
| Retinal hemorrhage | 1 (<0.1) | 0 | 1 (<0.1) |
| **Vascular disorders** | **2 (0.2)** | **1 (<0.1)** | **1 (<0.1)** |
| Aneurysm ruptured | 1 (<0.1) | 0 | 1 (<0.1) |
| Arterial hemorrhage | 1 (<0.1) | 1 (<0.1) | 0 |
| **Respiratory, thoracic and mediastinal disorders** | **1 (<0.1)** | **0** | **1 (<0.1)** |
| Hemoptysis | 1 (<0.1) | 0 | 1 (<0.1) |
| **Gastrointestinal disorders** | **28 (2.8)** | **12 (1.2)** | **16 (1.6)** |
| Diverticulum intestinal hemorrhagic | 1 (<0.1) | 0 | 1 (<0.1) |
| Duodenal ulcer | 1(<0.1) | 1 (<0.1) | 0 |
| Gastric hemorrhage | 2 (0.2) | 0 | 2 (0.2) |
| Gastrointestinal hemorrhage | 7 (0.7) | 2 (0.2) | 5 (0.5) |
| Hematemesis | 2 (0.2) | 2 (0.2) | 0 |
| Melena | 7 (0.7) | 2 (0.2) | 5 (0.5) |
| Lower gastrointestinal hemorrhage | 1 (<0.1) | 0 | 1 (<0.1) |
| Small intestinal hemorrhage | 4 (0.4) | 2 (0.2) | 2 (0.2) |
| Hemorrhagic ascites | 1 (<0.1) | 0 | 1 (<0.1) |
| Intra-abdominal hemorrhage | 3 (0.3) | 3 (0.3) | 0 |
| **Hepatobiliary disorders** | **1 (<0.1)** | **1 (<0.1)** | **0** |
| Hemobilia | 1 (<0.1) | 1 (<0.1) | 0 |
| **Renal and urinary disorders** | **3 (0.3)** | **0** | **3 (0.3)** |
| Hematuria | 2 (0.2) | 0 | 2 (0.2) |
| Renal hemorrhage | 1 (<0.1) | 0 | 1 (<0.1) |
| **Injury, poisoning and procedural complications** | **1 (<0.1)** | **0** | **1 (<0.1)** |
| Post procedural hemorrhage | 1 (<0.1) | 0 | 1 (<0.1) |

MedDRA version 23.0
